# The relationship between coping self-efficacy and B cells in breast cancer patients

**DOI:** 10.1186/s43046-023-00187-y

**Published:** 2023-09-04

**Authors:** Azza El-Amir, Eman M. El-Baiomy, Noha A. Sabry, Loay Kassem, Margaret A. Chesney, Kenneth A. Wallston

**Affiliations:** 1 Immunology Department, Faculty of Science, Cairo University, Cairo, Egypt; 2 Psychiatry Department, Faculty of Medicine, Cairo University, Cairo, Egypt; 3 Clinical Oncology Department, Faculty of Medicine, Cairo University, Cairo, Egypt; 4 Department of Medicine, University of California, San Francisco, CA USA; 5 Institute for Medicine and Public Health, Vanderbilt University Medical Center, Nashville, TN USA

**Keywords:** Coping self-efficacy, Depression, B cells, Immunity, DHEA, Breast cancer

## Abstract

**Background:**

Breast cancer is the most common tumor among women throughout the world. Diagnosis and treatment of breast cancer are associated with stress and depression. Self-efficacy is one of the most important personal characteristics, studied in cancer, and is correlated with depression and immunity. The aim of the study is as follows:

1. Examining the correlation between coping self-efficacy with depression, DHEA levels, and immunity

2. Examining the correlation between depression and DHEA levels

3. Studying the effect of depression and DHEA levels on immunity

4. Examining the intermediate effect of DHEA levels on the correlation between coping self-efficacy and immunity

**Methods:**

Thirty newly diagnosed breast cancer patients recruited from the Oncology Department, Kasr EL-Aini, Cairo University (ages 51.40 + 8.24 years) responded to two questionnaires: Coping Self-Efficacy Scale (CSES) and Patient Health Questionnaire-9 (PHQ-9); blood samples were collected to measure the phenotype of patients’ cellular immunity and DHEA levels by flowcytometry and ELISA technique.

**Results:**

There was a significant negative correlation between CSES and PHQ-9, a significant positive correlation between PHQ-9 and B-cell count, and there is a significant negative correlation between CSES and B-cell count. The presence of DHEA has no mediatory role on correlation between CSES and B-cell count.

**Conclusion:**

This paper presents a new model of psychoneuroimmunology by suggesting an effect of coping self-efficacy on immunity against breast cancer patients.

## Introduction

 Breast cancer is the second leading cause of death in women [1]. Breast cancer patients account for as much as 36% of cancer patients. An estimated 2089 million women were diagnosed with breast cancer in 2018 [2]. It has been reported that 156 out of 200 women with breast cancer had suffered from a shocking life event, usually the loss of a dear friend or person in her life. Other recent research has confirmed that breast cancer patients report distress that negatively affects their immunity [3, 4].

Psychology and immunology are interrelated as reflected in the development of psycho-oncology and psychoneuroimmunology, which have received increasing attention in recent years [5]. And the role of the hypothalamic-pituitary adrenal axis in studying psychoneuroimmunology has been documented [6, 7].

Previous cancer research has found a significant correlation between patients’ mental health status or personality traits and patient’s immunity against cancer. For example, research examining the correlation between locus of control and immune status in breast cancer patients confirmed that there is a positive correlation between high internal locus of control belief with natural killer cells (NK) cells and T-helper cells, and a significant inverse correlation was found between doctor health locus of control with NK, T helper, and Treg cells [8]. Another study of breast cancer patients found a significant negative correlation between God health locus of control belief and interleukin-6 (IL-6) [9].

One of the most important personal characteristics relevant to coping with the stress of being diagnosed with breast cancer is coping self-efficacy. Breast cancer patients with high coping self-efficacy have been shown to better manage their treatment side effects such as vasomotor symptoms, fatigue, pain, arthralgia, and neuralgia [10]. In breast cancer survivors, coping is very important in fighting cancer because it facilitates adaptation to illness [11].

Self-efficacy relates to one’s belief about the ability to perform coping behaviors such as stopping unpleasant thoughts when under stress [12]. High levels of self-efficacy decrease catecholamine secretion under stress [13]. A study of adolescents undergoing cancer treatment suggested that self-efficacy has a negative correlation with distress [14].

Another study suggested that higher levels of stress are related to low self-efficacy in college students [15]. A study on head and neck cancer patients suggested that high self-efficacy was significantly associated with less psychological distress [16].

These findings suggest that coping self-efficacy might be associated with dehydroepiandrosterone (DHEA) levels and immunity in breast cancer patients.

### Therefore, this study examined the following:

The correlation between coping self-efficacy with depression, DHEA levels, and immunity

1. The correlation between depression and DHEA levels
2. The effect of depression and DHEA levels on immunity
3. The intermediate effect of DHEA levels on the correlation between coping self-efficacy and immunity

## Materials and methods

### Breast cancer patients’ population

Thirty newly diagnosed breast cancer patients (ages 38–67) were recruited for participation in this research from the Department of Clinical Oncology, Kasr EL-Aini, Cairo University, from January 2022 to January 2023. Cairo University’s ethics committee approved the protocol of this research (code: N-70–2022) at 20 October 2022. The documented pathological proofs of breast cancer and the clinical data were available from the Department of Clinical Oncology’s database, and the demographic data are available in Table 1.

**Table 1:** Breast cancer patients’ demography

Variables	Patients (*n* = 30)	
	*n*	%
**Age in years**		
Range	38–67	
Mean ± SD	51.40 ± 8.24	
**Residence**		
Rural	9	30
Urban	21	70
**Educational level**		
Illiterate	14	46.7
General education	10	33.3
University education	6	20.0
**Job employed?**		
Not working	28	93.3
Working	2	6.7
**Marital status**		
Single	0	0.0
Married	20	66.7
Widow	5	16.7
Divorced	5	16.7

Inclusion criteria were as follows: (1) Early diagnosed female breast cancer patients (have not received any type of cancer therapy); (2) breast cancer stage (0, I, II, III); (3) age ranging from 25 to 70; and (3) written informed consent. Exclusion criteria were as follows: (1) Patients with a history of comorbid psychiatric disorders and (2) patients with diseases other than cancer that affect their immune status.

### Coping self-efficacy

Coping self-efficacy, defined as a belief about the ability of an individual to perform specific coping behaviors, was measured by the Coping Self-efficacy Scale (CSES) [17]. The CSES consists of 26 items that ask the participant to rate their confidence in his or her ability to cope effectively with a number of challenges. For example, participants are asked, “When things aren’t going well for you, or when you’re having problems, how confident or certain are you that you can do the following….” Then responses for each item were on 11-point scale: (0, cannot do at all), (5, moderately certain can do), and (10, certain can do).

The total score of the CSES is created by summing up the item’s rating. A higher score indicates higher coping self-efficacy [17]. The CSES has been widely used in studying coping self-efficacy in a range of patient populations. The CSES has good reliability (*r* = 0.92).

The permission to use Coping Self-efficacy Scale was obtained from the original authors.

### Patient Health Questionnaire (PHQ-9)

The Patient Health Questionnaire (PHQ-9) is a short self-report scale consisting of nine items to indicate the presence and symptoms severity of major depressive disorder [18]. PHQ-9 scale is the preferred screening tool, particularly in resource-limited settings, and it is recommended by the fifth edition of the *Diagnostic and Statistical Manual of Mental Disorders* [19].

Participants are asked to assess nine depressive symptoms items experienced by them over the last 14 days preceding the interview. The items share a common set of response options: (0, not at all), (1, several days), (2, more than half days), and (3, nearly every day). The total scores of the PHQ-9 scale range from zero to 27. Higher scores mean more severe depression [20].

The translation of the two scales was done by a rigorous translation approach; this involves three people who are highly familiar with Arabic and the English language.

### Biological measurements

A 3-cm peripheral venous blood sample was collected from all patients between 9 am and 12 pm after the two psychological scales were filled to control for diurnal variation. A total of 1.5 cm of blood sample kept in a plain tube was spun at 3000 g for 10 min at 4–8 °C. Separated serum was stored at − 80 °C until assay for DHEA concentration. A total of 1.5 cm of the blood sample was dispensed in ethylenediaminetetraacetic acid (EDTA) tubes and was used to assay patients’ immunity by flow cytometry. All blood specimens were de-identified and coded using study identification numbers.

### Serum dehydroepiandrosterone (DHEA)

Diametra ELISA kit (Boldn, UK) was used for immunoenzymatic determinations of serum DHEA on the TECAN analyzer (Switzerland) following the manufacturer’s specifications.

Each sample was measured in duplicate. The investigating lab has broad experience in performing these assays.

### Breast cancer patients’ cellular immunity

The immunity tests evaluated B cells: cluster of differentiation CD 20 + , total T cells: CD3 + , T-helper cells: CD4 + , CD3 + CD4 + , and cytotoxic T cells: CD8 + , CD3 + CD8 + by using CytoFLEX-flow cytometry instrument from Beckman Coulter (Brea, CA, USA). The immunophenotyping was performed on lymphocytes derived from viable white blood cells and CD45 gating. The antibodies used were CD3-PC5.5, CD4-FTC, CD8-PE, CD20-FITC, and CD45-PC7. Reagents were from Beckman Coulter. The staining of the cells by the antibody was made according to the manufacturer’s specifications.

### Statistical analysis

Quantitative variables were presented as range, mean, and standard deviation (SD). Qualitative variables were presented as frequency and percentage (%).

Pearson’s correlation coefficient was done to estimate the degree of correlation between two quantitative variables. Mediation model was conducted to estimate the effect of an independent variable on a dependent variable through a third explanatory one, known as a mediator variable.

Statistical analysis was done by SPSS v28 (IBM Inc., Chicago, IL, USA).

## Results

### Correlation between the CSES and PHQ-9 in breast cancer patients.

Table 2 shows the correlation between the CSES scores with PHQ-9, and the results show that there is a significant negative correlation between CSES with PHQ-9 scores.

**Table 2:** Correlation between CSES scores and PHQ-9 in breast cancer patients

	**PHQ-9**	
	***r***	***p***
**CSES**	− 0.316	0.018*

*CSES* Coping Self-Efficacy Scale, *r* correlation coefficient, *significant as *P*-value ≤ 0.05

### Correlation between CSES and PHQ-9 scores with DHEA in breast cancer patients

Table 3 studies the correlation between CSES and PHQ-9 with DHEA; the results show that there is no significant correlation.

**Table 3:** Correlation between CSES and PHQ-9 scores with DHEA in breast cancer patients

	**CSES**		**PHQ-9**	
	***r***	***P***	***r***	***P***
**DHEA**	− 0.059	0.665	0.119	0.382

*CSES* Coping Self-Efficacy Scale, *PHQ-9* Patient Health Questionnaire-9, *DHEA* dehydroepiandrosterone, *r* correlation coefficient

### Correlation between CSES and PHQ-9 with immunological parameters as a percentage of total lymphocytes in breast cancer patients

Table 4 is studying the correlation between CSES and PHQ-9 with immunological parameters (CD3 + T cells%, CD4 + T cells%, CD8 + T cells%, CD20 + B cells%, CD3 + CD8 + T cells%, and CD3 + CD4 + T cells%). The results show that there is a significant positive correlation between CSES and CD20 + B cells% and significant positive correlation between PHQ-9 and CD20 + B cells%.

**Table 4:** Correlation between the CSES and PHQ-9 with immunological parameters as a percentage of total lymphocytes in breast cancer patients

Immunological parameters	CSES		PHQ-9	
	***r***	***p***	***r***	***p***
CD3 + T%	0.055	0.686	− 0.069	0.615
CD4 + T%	− 0.168	0.215	0.106	0.438
CD8 + T%	0.192	0.156	− 0.243	0.071
CD20 + B%	− 0.292	0.029*	0.287	0.032*
CD3 + CD4 + T%	− 0.171	0.208	0.110	0.419
CD3 + CD8 + T%	0.167	0.220	− 0.138	0.311

*CSES* Coping Self-Efficacy Scale, *PHQ-9* Patient Health Questionnaire-9, *CD* cluster of differentiation, *r* correlation coefficient, *significant as *P*-value ≤ 0.05

### Correlation between DHEA and immunological parameters as a percentage of total lymphocytes in breast cancer patients

Table 5 is studying the correlation between DHEA and immunological parameters (CD3 + T cells%, CD4 + T cells%, CD8 + T cells%, CD20 + B cells%, CD3 + CD8 + T cells%, and CD3 + CD4 + T cells%). The results show that there is no significant correlation between DHEA and immunological parameters.

**Table 5:** Correlation between DHEA and immunological parameters as a percentage of total lymphocytes in breast cancer patients

Immunological parameters	DHEA	
	***r***	***P***
**CD3 + T%**	0.192	0.156
**CD4 + T%**	0.063	0.643
**CD8 + T%**	0.062	0.648
**CD20 + B%**	− 0.018	0.897
**CD3 + CD4 + T%**	0.065	0.632
**CD3 + CD8 + T%**	0.113	0.409

*DHEA* dehydroepiandrosterone, *CD* cluster of differentiation, *r* correlation coefficient, *significant as *P*-value ≤ 0.05

### *The intermediate role of DHEA in the correlation between the CSES and CD20* + *B cells% in breast cancer patients*

DHEA had an insignificant intermediate effect on the relationship between CSES and CD20 + B cells% as 0 was contained in the confidence interval.

Once the intermediate effect of DHEA was removed, CSES had a significant effect on CD20 + B cells% with a direct effect of − 0.037 (95% *CI*: − 0.069, − 0.004) (Table 6).

**Table 6:** Bootstrap indirect and direct effects analysis of nonparametric ratios

Mediating effect path		Effect	Standard error	Boot LLCI	Boot ULCI
CSES → DHEA → CD20%	Indirect effect	0.0003	0.003	− 0.007	0.006
CSES → CD20% after removing DHEA	Direct effect	− 0.037	0.016	− 0.069	− 0.004

*CSES* Coping Self-Efficacy Scale, *CD* cluster of differentiation, *DHEA* dehydroepiandrosterone, *LLCI* lower limit of 95% confidence interval, *ULCI* upper limit of 95% confidence interval

## Discussion

The correlation between psychological status and the efficiency of the immune system has been confirmed by many psychoneuroimmunology studies [21]. The results of this study are consistent with the psychoneuroimmunology framework by suggesting a negative correlation between CSES and CD20 + B cells% in breast cancer patients.

Our study proposed the following hypotheses:

- 1) There is a correlation between CSES and PHQ-9 scores.
- 2) CSES, PHQ-9, and DHEA effect on immunological parameters (CD3 + T cells%, CD4 + T cells%, CD8 + T cells%, CD20 + B cells%, CD3 + CD8 + T cells%, and CD3 + CD4 + T cells%).
- 3) DHEA mediated the correlation between CSES with immunological parameters.

Regarding the first hypothesis that there is a significant relationship between CSES and PHQ-9, our results confirmed that there is a negative correlation between CSES and PHQ-9. Thus, patients with high coping self-efficacy have fewer depression symptoms than patients with low coping self-efficacy. Our results are consistent with a research study on coronary heart disease which suggested that depressed mood was associated with lower perceived health competence (*a* =  − 0.21, *p*< 0.001) [22]. A study of melanoma patients suggested that increasing self-efficacy for coping with cancer decreased symptoms of depression among cancer patients [23]. Also, our study is consistent with a study of doctoral students that found a negative correlation between self-efficacy and depression [24], and another study on newly HIV diagnosed which confirmed that higher general self-efficacy was associated with lower levels of depression [25].

Regarding the second hypothesis that CSES, PHQ-9, and DHEA effect on immunological parameters (CD3 + T cells%, CD4 + T cells%, CD8 + T cells%, CD20 + B cells%, CD3 + CD8 + T cells%, and CD3 + CD4 + T cells%). Our results show that there is a significant positive correlation between PHQ-9 and CD20 + B cells% and negative correlation between CSES and CD20 + B cells%. DHEA has no significant effect on immunological parameters. Our result is not consistent with two studies on male breast cancer and prostate cancer made by *Yang and his colleagues*in 2018 using a Brief version of cancer behavior inventory to measure patients’ self-efficacy and suggested that there is no significant correlation between self-efficacy and CD19 + B cells [26, 27]. These studies, however, did not use the CSES to assess self-efficacy but rather relied on a brief version of the Cancer Behavior Inventory. Using different scales may be the reason for different results. Also, our results are consistent with two studies in human depression that have shown reduced IL-10 and producing regulatory B cells in patients compared with nondepressed controls [28, 29].

Regarding the third hypothesis that DHEA would mediate the correlation between the CSES with immunological parameters, our results rejected this hypothesis. There is a significant negative correlation between scores on the CSES and B cells. But the presence of DHEA converts this correlation to non-significant. According to our knowledge, there is no previous research that studied the role of DHEA on mediating the correlation between coping self-efficacy and immunological parameters. More research is recommended to explain the mechanism of correlation between CSES scores and immunological parameters.

## Conclusion

B cells may have pro- or anti-tumorigenic responses in the tumor microenvironment as there is a heterogeneous population of B cells with different functions. Our research suggested a decreasing effect of CSES on B cells. And this relationship presents new model of psychoneuroimmunology.

## Data Availability

The datasets used and/or analyzed during the current study are available from the corresponding author on reasonable request.

## Abbreviations

*****: Significant as *P*-value ≤ 0.05
CD: Cluster of differentiation
CSES: Coping Self-efficacy Scale
DHEA: Dehydroepiandrosterone
EDTA: Ethylenediaminetetraacetic acid
IL-6: Interleukin-6
LLCI: Lower limit of 95% confidence interval
NK: Natural killer cells
PHQ-9: Patient Health Questionnaire
r: Correlation coefficient
SD: Standard deviation
ULCI: Upper limit of 95% confidence interval
